# Adherence to the EAT–Lancet Diet: Unintended Consequences for the Brain?

**DOI:** 10.3390/nu14204254

**Published:** 2022-10-12

**Authors:** Hayley Anne Young

**Affiliations:** Faculty of Medicine, Health, and Life Science, Swansea University, Swansea SA2 8PP, UK; h.a.young@swansea.ac.uk

**Keywords:** sustainability, EAT–Lancet diet, cluster analysis, heart rate variability, mood, cognition

## Abstract

In January 2019, the EAT–Lancet Commission defined a universal reference diet to promote human and environmental health. However, in doing so, the potential consequences for brain health were not considered. Whilst plant-based diets are generally associated with better cognitive and affective outcomes, those that severely limit animal products are not. Therefore, the potential ramifications of the EAT–Lancet diet on cognition, mood, and heart rate variability were considered (*N* = 328). Adherence to the Alternative Healthy Eating Index (AHEI) was associated with having a better mood, focused attention, working and episodic memory, and higher heart rate variability. However, when the EAT–Lancet diet was considered, the effects were either smaller or not significant. Cluster analysis identified a dietary style characterised by a strong adherence to the EAT–Lancet recommendation to limit meat intake, representing a sixth of the present sample. This group had a lower Mean Adequacy Ratio (MAR); did not meet the Recommended Nutrient Intake (RNI) for a range of nutrients including protein, selenium, zinc, iron, and folate; and reported a poorer mood. These data highlight the potential unintended consequences of the EAT–Lancet recommendations for nutritional adequacy and affective health in some individuals. There is a need to better optimise the EAT–Lancet diet to support brain health. As we move towards more sustainable diets, these findings emphasise the need to consider how such diets might affect the brain.

## 1. Introduction

Current diets are negatively influencing both planetary and human health [1]. Therefore, altering diets so that they are more sustainable is high on the global political agenda [2]. However, it is crucial to ensure that dietary changes designed to reduce the detrimental effects of human activity on the planet do not have unintended consequences for human health. Recently, the EAT–Lancet Commission proposed a global reference diet representing the current evidence base for a healthy and an environmentally sustainable diet [1]. The diet included mainly plant-based foods, limited dairy products, and very little meat. The EAT–Lancet Commission estimated that this diet would prevent 19–24% of total deaths globally [1]. However, the human health benefits of the EAT–Lancet diet, compared to a standard healthy diet, remain controversial [3,4,5,6]. In particular, the EAT–Lancet diet has not yet been evaluated regarding its effects on the brain. Therefore, we examined the association between the EAT–Lancet diet and cognition, mood, and heart rate variability: a biomarker suggested to reflect general psychological and physiological health [7].

Generally, dietary patterns with more plant-based foods and a lower consumption of meat, such as the Mediterranean diet, have been associated with better health [8], including metabolic, cardiovascular, and cognitive health [9,10,11,12]. Such effects may be attributed to a higher intake of plant-based foods rich in vitamins, minerals, and antioxidants and/or a lower intake of red and processed meats [9,10,11,12]. Importantly, similar benefits were observed using dietary pattern scores designed to approximate the EAT–Lancet diet: higher adherence to the EAT–Lancet diet was associated with lower risks of ischaemic heart disease and diabetes [13], stroke [14], and overall mortality [15], although effects on cognition or mood have not yet been examined.

Conversely, it is important to consider that not all plant-based diets are equally healthy. For example, a reduction in animal products may exclude beneficial food groups such as dairy and fish, resulting in diets that are low in n-3 PUFA, proteins, calcium, zinc, iron, and vitamins B12 and D [16,17]. Indeed, the original EAT–Lancet diet score developed by Knuppel and Papier [13] used minimum intake values for various dietary components set at 0 g/day, including meat, fish, and dairy. Subsequently, it was observed that using minimum intake values of 0 g/day for these food groups was associated with an increased risk of deficiency and a decreased probability of micronutrient adequacy, including calcium, folate, iron, vitamin A, vitamin C, and zinc, particularly amongst women of childbearing age [18]. In addition, vegan diets, which exclude all animal products, were associated with a low intake of vitamins B_2_, niacin (B_3_), B_12_, D, iodine, zinc, calcium, potassium, selenium, and protein [19]. This is concerning, as an inadequate intake of these nutrients was associated with poorer mood [20,21,22], cognitive functioning [21,23,24], and a predisposition towards cognitive decline [25,26,27]. The adequate intake of these nutrients is particularly crucial during pregnancy to regulate neurodevelopment [28]. Deficiency during this time was associated with potential lifelong consequences for both cognition and mood [28,29].

Notably, in developing the reference diet, the EAT–Lancet Commission considered evidence from intervention trials and long-term observational studies that related individual dietary components and overall dietary patterns to major disease endpoints such as cancer, cardiovascular disease, diabetes, and overall mortality [1]. Although cognition was briefly mentioned in relation to fish, fruit, and vegetable intake, consideration of the psychological literature was, in general, lacking. In addition, any consideration of effects on mood or mental health was absent. Notably, a recent expert group evaluated the potential cognitive effects of the EAT–Lancet diet, concluding that current evidence was limited [30]. Therefore, the empirical evaluation of the effects of the EAT–Lancet reference diet on cognitive and affective health is crucial.

Furthermore, when the healthiness of the reference diet was assessed for its nutrient adequacy and nutrient composition, it was concluded that adopting the diet would improve nutrient intake for most nutrients, including iron, zinc, folate, vitamin A, and calcium, although supplementation with B12 might be necessary [1]. However, whilst it is theoretically possible to achieve nutrient-dense sustainable diets, this requires the consumption of a combination of complementary ‘alternative’ proteins, such as wheat, nuts, legumes, and soy. However, combining quantities of different food items to optimise specific goals, i.e., health and sustainability, can result in recommended diets that are far from what people are willing to eat. Therefore, an important step is determining the nutritional adequacy of sustainable dietary patterns within the constraints of cultural acceptability. In other words, within the bounds of what a given population would normally consume.

Similarly, so far, the association between the EAT–Lancet diet and health outcomes have only been considered using a range of a priori composite dietary pattern scores calculated to represent the reference diet [13,14,15,31,32]. However, a disadvantage of this approach is that more than one scenario can lead to similar total scores. For example, consuming a significant amount of the more desirable food groups (e.g., fruit and vegetables), along with a significant amount of a less desirable food (e.g., red meat), can give the same score as consuming a moderate amount of each (31). In this context, data-driven approaches such as cluster analysis, which is used to create groups of people with homogenous dietary patterns, may give a more nuanced view of specific dietary patterns that exist within planetary health boundaries, and how those patterns relate to health outcomes. Such approaches have not yet been applied to sustainable diets but have been successfully used to identify healthy and unhealthy patterns within the vegan diet [33].

In summary, there is an urgent need to shift current dietary consumption patterns towards those that are more sustainable. In 2019, the EAT–Lancet Commission on healthy diets from sustainable food systems described a healthy reference diet, designed to be environmentally sustainable whilst also preventing diet-related chronic diseases and mortality [1]. However, it is not yet clear whether the diet will benefit cognitive and affective health. In addition, the healthiness of the diet has not yet been evaluated, taking into consideration the everyday food that people who eat sustainably are prepared to consume.

Therefore, this study had two aims: (1) to determine for the first time the association between the EAT–Lancet diet and cognition, mood, and heart rate variability and (2) identify healthy and unhealthy dietary patterns that are environmentally sustainable. Given that plant-based diets are generally associated with better cognitive health [34], the hypothesis was that adherence to the EAT–Lancet diet would be associated with cognition, mood, and heart rate variability. However, as observed with the vegan diet [19,33], it was anticipated that some individuals who adhere strongly to a planetary health diet may consume inadequate quantities of certain nutrients, which could have consequences for the brain.

## 2. Materials and Methods

### 2.1. Procedure

This was a secondary analysis of data collected for a range of studies between 2015 and 2019 [35,36,37,38,39,40,41,42]. When datasets included more than one electrocardiogram (ECG) measurement, for example, before/after an intervention, only baseline measurements were included. In each study, participants were asked not to consume alcohol or take part in exercise for twenty-four hours before the study. They were also asked to fast for at least two hours before attending the laboratory, although some studies included an overnight fast [36,37,40,41]. Preliminary analysis indicated that the duration of the fast did not influence the results.

Participants were recruited either through word of mouth, advertising on social media (e.g., Facebook, Reddit), or through the Swansea University psychology participant pool. On entering the laboratory, written informed consent was provided and the participants completed a questionnaire pack that included the European Prospective Investigation into Cancer and Nutrition Norfolk Food Frequency Questionnaire (FFQ) [43]; the profile of mood states (POMS) [35,36,37,38,40,41,44], or the positive and negative affect scale (PANAS) [39,45]—these data were retrospectively harmonised according to published guidelines [46,47].

Following this, participants’ height and weight were measured and were fitted either with a RS800 Polar heart rate monitor electrode transmitter belt (T61) using conductive gel (Polar Electro, Kempele, Finland) [35,36,37,38,41] or Ag/AgCl electrodes and transducers connected to a BIOPAC MP150 and ECG100C amplifier module (BIOPAC, Goleta, CA, USA) [39,40,42]. In all studies, R–R interval data were recorded whilst participants rested quietly in a semi−supine position for six minutes. Finally, participants completed the test battery detailed below. All procedures were approved by the Swansea University ethics committee and carried out in accordance with the principles laid down by the Declaration of Helsinki (2013). A schematic illustration of the procedure can be found in Supplementary Information: Figure S1.

### 2.2. Participants

Power analysis (G power) used effect sizes (small to medium) previously found when relating habitual diet quality and heart rate variability (HRV)/mood [38]. It was estimated that an expected effect size of *r* = 0.25, α < 0.05, and 80% power would require 120 participants. In the event, after exclusions detailed below, the sample included 206 females and 122 males between 18 and 35 years of age who had complete heart rate variability and mood data. With cognition, there was a sample available of 91 females and 69 males who were between 18 and 35 years of age, indicating that we had adequate power for our a priori analysis. In all studies, participants were excluded at the point of recruitment if they had a cardiovascular or metabolic disorder (e.g., diabetes); gastrointestinal problems (e.g., Crohn’s disease); were pregnant; or were diagnosed with a mood (e.g., depression), developmental (e.g., ADHD/autism), or eating (e.g., bulimia nervosa) disorder. Medications/supplements were recorded and those taking medications or herbal supplements to control appetite and/or body weight were excluded. As we were interested in the effects of diet, participants taking nutritional supplements, e.g., multivitamins, omega 3, etc., were also excluded retrospectively, as were a relatively small number of smokers and/or vapers (*n* = 18). Body mass index (BMI) ranged from 17.8 to 37.04 kg/m^2^.

### 2.3. Habitual Diet

Food frequency data were collected using the European Prospective Investigation into Cancer and Nutrition Norfolk Food Frequency Questionnaire (EPIC-Norfolk FFQ) [43]. After a specific portion size was specified, participants were asked to indicate on a nine-point scale (0 = ‘never’ to 9 = ‘6+ per day’) how often they tended to consume specific foods. The EPIC-Norfolk FFQ has been previously validated by comparing it with a 16-day weighed food record [48] and nutrient biomarkers [49]. The FETA software, based on UK food-composition databases [43], was used to ensure food groups were captured cleanly. For example, various proportions of ‘fruit juice’ count towards added sugars, whilst the remaining fractions count toward total fruits. Crucially, alternative proteins not captured on the EPIC-Norfolk FFQ (e.g., whey protein) were captured by asking participants to specify these under ‘other foods’ and detail their frequency of consumption. These were manually transformed using McCance and Widdowson’s ‘composition of foods integrated dataset [50] and added to the FETA composition scores. From the resulting nutrient/food group, a modified version of the alternate healthy eating index (AHEI) score [51] and a world index for sustainability and health (WISH) score [31] were created (supplementary information).

#### 2.3.1. Alternate Healthy Eating Index 2010 (AHEI-2010) Score

The AHEI-2010 provides a measure of diet quality that is used to identify the future risk of diet-related chronic disease [52] and has been previously associated with mood, HRV, and cognition [38,41,53]. We chose this score so that our findings were comparable to those from other UK-based cohort studies that have examined the association between diet and psychological health. The AHEI was calculated by taking the sum of 6 protective component scores (1: fruit; 2: vegetable; 3: whole grains; 4: nuts and legumes; 5: long-chain ω-3 fatty acids; 6: PUFA excluding long chain n-3), and 4 dietary components that should be limited (1: red/processed meat; 2: sugar-sweetened beverages; 3: trans fatty acids; 4: sodium). Further details are available in Supplementary Table S1. Note that, whilst the AHEI-2010 considers alcoholic drinks, these were not included, as they are not generally included in planetary health diet scores [13,31,32]. Therefore, for consistency, alcohol was considered a covariate in the main analysis. A higher AHEI score corresponded to a healthier diet.

### 2.3.2. World Index for Sustainability and Health (WISH)

The world index for sustainability and health (WISH) score [31] was based on the EAT–Lancet reference diet. This index was chosen as, unlike other planetary heath indices [13], it does not limit the possible quantities of some food groups, such as fruit and vegetables, for which there is no upper limit on intake. Additionally, other planetary health scores are based on caloric density [32]. However, taking a caloric-density approach leads to associations between dietary scores and health outcomes that are difficult to interpret independently of total calories in the diet [54]. Therefore, total calories in the diet was instead considered a covariate. The pro and cons of each approach are debated, and the interested reader is referred to Tomova, Arnold [54], for a review of the topic.

Specific details regarding the computation of WISH can be found in supplementary information. Briefly, thirteen food groups (1: whole grains; 2: vegetables; 3: fruits; 4: dairy foods; 5: red meat; 6: fish; 7: eggs; 8: chicken and other poultry; 9: legumes; 10: nuts; 11: unsaturated oils; 12: saturated oil; 13: added sugars). are scored according to their healthiness (protective, limited, neutral) and impact on the environment (high, medium, low) (Supplementary Table S2). For the calculation of the total WISH score, all food group components are summed up and are given equal weight (a higher score indicates a healthier, environmentally friendlier diet).

### 2.4. Heart Rate Variability

Kubios HRV Analysis Software 3.4.0 (The Biomedical Signal and Medical Imaging Analysis Group, Department of Applied Physics, University of Kuopio, Kuopio, Finland) [55] was used to analyse R–R data.

#### Pre-Processing

Initially data were visually inspected for ectopic beats, poor conductivity, etc., which may distort the variability analysis [35]. In the event, 2 cases were removed due to poor recording. Artifacts were then corrected using a very low correction threshold (0.45 from local average), and less than 1.2% of beats were identified as artefacts.

Time-series data were transformed into the frequency domain using the fast Fourier transform (FFT). Cubic spline interpolation at a rate of 4 Hz was used to create equidistant samples, and Welsh’s periodogram was used to divide the R–R series into overlapping windows to decrease the leakage effect. Vagal activity was quantified by estimating the average spectral power within the high-frequency (0.15–0.4) band.

### 2.5. Cognition and Mood

#### 2.5.1. Episodic Memory: Word-List Recall

Fifteen abstract and fifteen concrete words, matched for the number of syllables, imageability, and frequency, were selected from the MRC Psycholinguistic Database. Participants listened to a recording of the words, which were presented at a rate of one word every two seconds. Short-term recall was assessed by asking participants to write down as many words as they could remember immediately after presentation. Delayed recall was assessed by asking participants to again write down as many words as they can remember, this time after the completion of the other tasks (25 min later).

#### 2.5.2. Working Memory

Working memory was assessed using a computerised version of the serial sevens test. Starting from a randomly generated number between 800 and 999, participants had to say whether a subsequent given number was seven less than the number that came previously. There were 28 trials. The outcome measures were the percent correct and the average time to respond in milliseconds.

#### 2.5.3. Focused Attention

Selective attention was assessed using the Eriksen and Eriksen (1974) flanker task. Each trial presented five arrows in the centre of the screen that were either congruent (>>>>>), incongruent (>><>>), or neutral (□□<□□). Participants were required to focus their attention on the middle arrow and to indicate using the keyboard whether it was pointing to the left or to the right. There were 72 trials, each with a randomly varying inter-stimulus interval of between 1 and 3 s. The outcome measures were percent correct for each type of trial (congruent, incongruent, neutral) and mean reaction time in milliseconds.

#### 2.5.4. Depressed Mood

Mood was assessed either using the profile of mood states (POMS) [35,36,37,38,40,41,44] or the positive and negative affect scale (PANAS) [39,45]. Using the 12-item elated—depressed subscale of POMS, participants rated each item on a scale 0–3 (0 ‘not at all’, 3 ‘a lot like this’) how much they had felt that way in the past week, including today. A lower score indicated a more depressed mood. Similarly, the PANAS negative affect scale comprises 10 items that measure the degree of negative affect on a 5-point Likert-type scale. Cronbach’s alpha for the POMS and the PANAS were 0.89 and 0.86, respectively. To produce a single measure of depressed mood, these data were retrospectively harmonised according to published guidelines [46,47]. Specifically, harmonisation was achieved by collapsing scores into quantiles [47]. The lowest-scoring group was defined as having the best mood, and the highest-scoring group was defined as having the highest levels of negative affect.

### 2.6. Additional Covariates

#### 2.6.1. Body Mass Index (BMI)

An electronic scale (Kern KMS-TM, Kenr and Sohn GmbH, Germany) was used to measure body weight, and a portable stadiometer was used to measure height. BMI was calculated using the standard formula: weight (in kilograms)/height (in metres)^2^.

#### 2.6.2. Physical Activity

Participants reported the frequency and duration that they took part in a range of moderate and vigorous activities such as walking, cycling, fitness classes, gardening, housework, etc. The amount of physical activity was quantified by computing the total amount of time (min/day) spent at > 3.0 METs (i.e., moderate intensity).

### 2.7. Statistical Analysis

The distribution of each variable was visually inspected using histograms, and skewed distributions were corrected using a logarithmic transform. Paired sample *t* tests were used to determine sample adherence to the WISH diet, i.e., recommended food-group intake was compared with actual food-group intake. Frequencies were used to describe the percentage of the population adhering to recommended intakes.

Hierarchical multiple regression (SPSS version 26) was used to assess the association between diet and HRV/mood/percentage accuracy for the working-memory task, after controlling for the other demographic and lifestyle factors (sex, BMI, total calories, alcohol, and exercise). To determine the effects of diet on depressed mood/HRV/percentage accuracy over and above these factors, diet was then entered in the second step. The influence of the AHEI and WISH was considered in separate analysis. The data reported are standardised coefficients and 95% confidence intervals.

A 2 (immediate/delayed) × 2 (concrete/abstract) repeated measures ANOVA was used to determine the effect of diet on episodic memory. Similarly, a 3-way (congruent/neutral/incongruent) repeated-measures ANOVA was used to determine the effect of diet on attention reaction times and attention percentage accuracy. In all analyses, diet was entered as a continuous factor, and the covariates were sex, BMI, total calories, alcohol, and exercise. The influence of the AHEI and WISH were again considered in separate analyses.

For all analyses, potential outliers were detected using Cook’s (1977) distance with a threshold of 4/N. Cases exceeding this threshold were excluded. Benjamini and Hochberg’s (1995) false-discovery rate (FDR) was employed and controlled at δ = 0. Where relevant, the Greenhouse–Geisser correction was used to correct degrees of freedom when Mauchly’s test of sphericity was significant.

### 2.8. Cluster Analysis

As an environmentally sustainable diet is not necessarily a healthy diet, we used K-means cluster analysis to identify sub-groups of individuals within the highest tertile of the EAT–Lancet diet. The K-means cluster analysis is based on Euclidean distances. The analysis requires the a priori specification of the expected number of clusters. This was unknown prior to analysis; therefore, the analysis was initially conducted with 2–6 clusters, and the best cluster solution was chosen considering the amount of variation explained by the solution, the screeplot, and the size and interpretation of each cluster. Once clusters were identified, differences between the clusters regarding food subgroups and nutrients were examined using *t* and Chi-squared tests, respectively. ANOVA was used to establish differences between clusters regarding mood and heart rate variability. In the event, we were unable to determine differences in cognition due to a limited sample size.

## 3. Results

### 3.1. Sample Adherence to the Planetary Health Diet (WISH)

**Figure 1:**
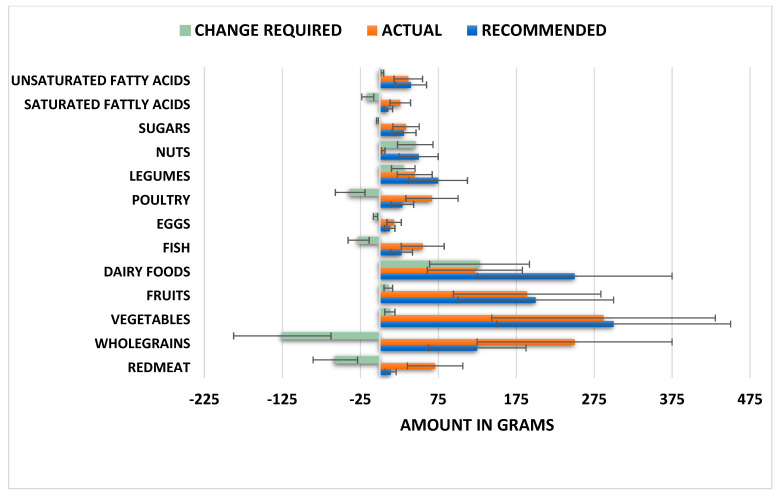
Diet gap between the actual intake and the recommended reference diet intake of food. When a range is recommended, average recommended intake is used. Data are mean (SEM).

As can be seen in Figure 1, the present population significantly overconsumed red meat (*t* (327) = 15.844, *p* < 0.001), whole grains (*t* (327) = 17.360, *p* < 0.001), fish (*t* (327) = 4.429, *p* = *p* < 0.001), poultry (*t* (327) = 11.759, *p* < 0.001), and saturated fatty acids (*t* (327) = 25.183, *p* < 0.001). Under consumed food included dairy products (*t* (327) = −39.224, *p* < 0.001), nuts (*t* (327) =−136.988, *p* < 0.001), legumes (*t* (327) = −13.058, *p* < 0.001), eggs (*t* (327) = −6.011, *p* < 0.001), and unsaturated fats (*t* (327) = −3.992, *p* < 0.001). Participants slightly under consumed vegetables (*t* (327) = −4.763, *p* < 0.001) and were consuming the recommended intake of both fruit (*t* (327) = 1.609, *p* = 0.109) and sugars (*t* (327) = 1.496, *p* = 0.136).

Notably, 71% of the sample did not adhere to the intake recommendations for red meat, and 66% of the sample did not adhere to those for poultry. A total of 96% of the sample consumed less than the minimum recommended intake for nuts, and 100% of the sample consumed <50% of the upper recommended intake for dairy. Thus, there is scope to increase the consumption of these under consumed foods. Interestingly, on average fruit and vegetable consumption were adequate.

However, 23% of the sample consumed less than the lower recommended intake for vegetables, and 31% consumed less than the recommended intake for fruit (Figure 1).

### 3.2. Associations between the EAT-Lancet Diet (WISH) and Health Outcomes

#### 3.2.1. Heart Rate Variability

Three potential outliers were identified; however, as removing these cases did not influence the results they were retained. The first model was significant (R^2^ = 0.051, F (5,325) = 3.410, *p* < 0.005): as expected, a higher physical-activity level was associated with a higher HRV (β = 0.261, LL 0.002, UL 0.013). However, BMI (β = 0.063, LL −0.221, UL 0.815), total kcal (β = −0.078, LL −0.012, UL 0.005), alcohol (β = −0.065, LL −0.403, UL 0.120), and gender (β = −0.064, LL −9.015, UL 2.452) did not contribute significantly to the model.

##### EAT-Lancet Diet (WISH)

The addition of WISH to the model did not significantly increase the amount of variance that could be explained (R^2^ change = 0.010, F (1,319) = 3.389, *p* < 0.067): there was no effect of the planetary health diet on HRV (β = 0.103, LL −0.010, UL 0.288).

##### Alternative Healthy Eating Index (AHEI)

Interestingly, in step 2, the addition of the AHEI significantly increased the amount of variance that could be explained (R2 change = 0.025, F (1,319) = 8.456, *p* < 0.004): those who adhered more closely to the dietary guidelines diet had higher HRV (β = 0.163, LL 0.156, UL 0.808).

#### 3.2.2. Mood

The first model was not significant (R^2^ = 0.022, F (5,327) = 1.463, *p* = 0.201). However, those who took more exercise reported having a less-depressed mood (β = −0.210, LL −0.001, UL −0.002). No other effects were significant: alcohol (β = −0.069, LL −0.023, UL 0.006), BMI (β = −0.010, LL −0.032, UL 0.027), sex (β = 0.082, LL −0.089, UL 0.556), calories (β = 0.134, LL −0.001, UL 0.001).

##### EAT-Lancet Diet (WISH)

The addition of WISH to the model significantly increased the amount of variance that could be explained (R^2^ change = 0.022, F (1,321) = 7.296, *p* < 0.007): those who adhered more closely to the planetary health diet had a less depressed mood (β = −0.153, LL −0.020, UL −0.003).

##### Alternative Healthy Eating Index (AHEI)

Effects were larger with the addition of the AHEI. The AHEI significantly increased the amount of variance in mood that could be explained (R^2^ change = 0.051, F (1,321) = 17.677, *p* < 0.001): those who adhered more closely to the dietary guidelines diet had a better mood (β = −0.057, LL −0.001, UL −0.021).

#### 3.2.3. Immediate and Delayed Episodic Memory

As expected, there was a main effect of delay (*F* (1, 157) = 9.702, *p* < 0.002, η^2^ = 0.058); participants remembered fewer words after a delay. There was also a delay X type interaction (*F* (1,157) = 7.258, *p* < 0.008, η^2^ = 0.044); participants forgot more abstract words after a delay. There was a significant delay X type X exercise interaction (*F* (1,157) = 5.126, *p* < 0.025, η^2^ = 0.032); those who engaged in more physical activity had better immediate concrete memory (r (157) = 0.219, *p* < 0.005), although the effect was larger after a delay (r (157) = 0.312, *p* < 0.001). No other main effects or interactions were significant.

##### EAT-Lancet Diet (WISH)

The delay X type X WISH interaction was not significant (*F* (1,157) = 1.773, *p* = 0.185, η^2^ = 0.011). Neither was the main effect of the WISH (*F* (1,157) = 0.599, *p* = 0.440, η^2^ = 0.004).

##### Alternative Healthy Eating Index (AHEI)

The delay X type X AHEI interaction did not reach significance (*F* (1,157) = 0.285, *p* = 0.594, η^2^ = 0.002). However, the main effect of the AHEI was significant (*F* (1,157) = 5.791, *p* < 0.017, η^2^ = 0.036). Participants who consumed a healthier diet had better memory (r (157) = 0.189, *p* < 0.017).

#### 3.2.4. Working Memory Reaction Times

Five outliers with a Cook’s distance >4/N were identified and removed. Upon inspection it was clear that these cases failed to respond appropriately to the task. Those who were male tended to have shorter reaction times (*F* (1,151) = 4.288, *p* < 0.036, η^2^ = 0.029). In addition, consuming more calories was associated with longer reaction times (*F* (1,151) = 4.068, *p* < 0.045, η^2^ = 0.026). No other covariates were significant.

##### EAT-Lancet Diet (WISH)

There was no effect of adherence to the planetary health diet on working memory reaction times (*F* (1,151) = 0.029, *p* = 0.886, η^2^ = 0.000).

##### Alternative Healthy Eating Index (AHEI)

Similarly, the effect of AHEI did not reach significance (*F* (1,151) = 2.164, *p* = 0.143, η^2^ = 0.014).

#### 3.2.5. Working Memory Accuracy

Those who consumed more calories made fewer errors (β = −0.310, LL −0.011, UL −0.001), although there was no effect of BMI (β = −0.011, LL −0.378, UL 0.326), sex (β = 0.072, LL −2.075, UL 5.557), alcohol (β = 0.044, LL −0.129, UL 0.222), or physical activity (β = 0.100, LL −0.002, UL 0.005).

##### EAT-Lancet Diet (WISH)

There was a trend towards those adhering to the planetary health diet producing fewer errors, but the effect was not significant (β = 0.145, LL −0.004, UL 0.196).

##### Alternative Healthy Eating Index (AHEI)

Adherence to the AHEI was associated with making fewer errors (β = 0.266, LL 0.174, UL 0.598).

#### 3.2.6. Focused Attention Reaction Times

As expected, there was a main effect of type of trial (*F* (2, 300) = 10.611, *p* < 0.001, η^2^ = 0.066); participants performed the task more slowly when distractors were incongruent. There was also a significant trail X sex interaction (*F* (2, 300) = 3.056, *p* < 0.049, η^2^ = 0.020), although follow-up tests did not reveal any differences. No other main effects or interactions reached significance.

##### EAT-Lancet Diet (WISH)

The interaction WISH X type of trial was not significant (*F* (2, 300) = 0.900, *p* = 0.407, η^2^ = 0.006), and neither was the main effect of WISH (*F* (1, 150) = 0.072, *p* = 0.789, η^2^ = 0.000).

##### Alternative Healthy Eating Index (AHEI)

There was no significant effect of AHEI: AHEI X type of trial interaction (*F* (2, 300) = 1.147, *p* = 0.319, η^2^ = 0.008), main effect (*F* (1, 150) = 0.029, *p* = 0.866, η^2^ = 0.000).

#### 3.2.7. Focused Attention Accuracy

Mauchly’s test of sphericity was significant (*χ*^2^(2) = 53.338, *p* < 0.001); therefore, degrees of freedom were corrected using Greenhouse–Geisser estimates of sphericity (ε = 0. 77). As expected, there was a main effect of the trial (*F* (1.539, 232.340) = 5.595, *p* < 0.011, η^2^ = 0.034); participants were less accurate on the incongruent trials. In addition, there was a significant exercise X trial interaction (*F* (1.539, 232.340) = 5.474, *p* < 0.009, η^2^ = 0.035), indicating that participants who took the least exercise performed less accurately on incongruent trials (r (157) = 0.173, *p* < 0.033). No other effects were significant.

##### EAT-Lancet Diet (WISH)

The trial X WISH interaction did not reach significance (F (1.539, 232.340) = 3.025, *p* = 0.064, η^2^ = 0.020), although participants who scored higher on the WISH performed more accurately but only on incongruent trials (r (157) = 0.325, *p* < 0.001).

##### Alternative Healthy Eating Index (AHEI)

Again, the trial X AHEI interaction reached significance (F (1.539, 232.340) = 11.277, *p* < 0.001, η^2^ = 0.070). Those who adhered more to the AHEI performed more accurately but only on incongruent trials (r (157) = 0.196, *p* < 0.015).

### 3.3. Identification of Healthy and Unhealthy Sustainable Dietary Patterns

**Figure 2:**
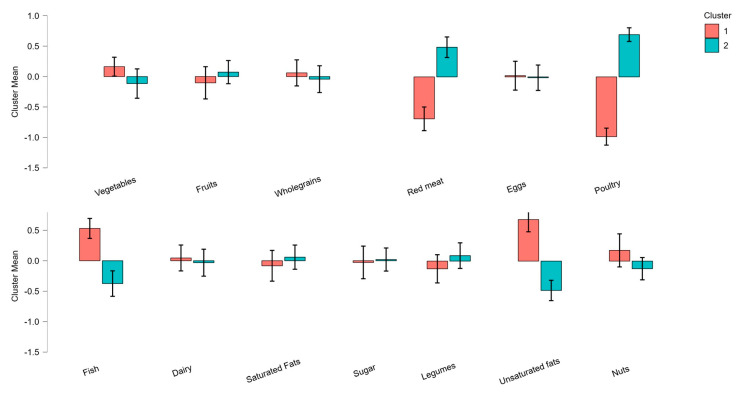
Cluster means for each WISH dietary component. Data are standardised means (95% CI).

Cluster analysis identified two dietary styles within the top tertial of the EAT–Lancet reference diet (Figure 2). Both clusters of individuals had high adherence to the EAT–Lancet recommendations for the low environmental impact foods (i.e., vegetable wholegrains, and added sugar); moderate adherence to recommendations for fruit, legumes, dairy, eggs, and saturated fats; and low adherence to the recommendation for nuts. However, whereas those in cluster 1 (*n* = 54; balanced diet) adhered strongly to the recommendation to consume moderate amounts of fish and unsaturated fats, they did not adhere to the recommendation to limit poultry and red meat. Conversely, cluster 2 (*n* = 57; restricted diet) scored lower in their adherence to fish and unsaturated oils but adhered strongly to the recommendation to limit poultry and red meat. Note that cluster 2 was not necessarily a vegetarian or vegan diet, as very small amounts of meat were consumed.

Those in cluster 2 were also more likely to be female (X^2^ (1,111) = 8.738, *p* < 0.003) and to consume fewer calories (*F* (1, 109) = 3.992, *p* < 0.048, η^2^ = 0.036). Those in cluster 1 tended to consume more alcohol (*F* (1, 109) = 3.772, *p* = 0.055, η^2^ = 0.034). Clusters did not differ regarding physical activity (*F* (1, 109) = 2.726, *p*  = 0.102, η^2^ = 0.025) or BMI (*F* (1, 109) = 0.136, *p* = 0.713, η^2^ = 0.001) (Table 1).

### 3.4. Association between Healthy and Unhealthy Sustainable Dietary Patterns and Nutrient Adequacy

**Figure 3:**
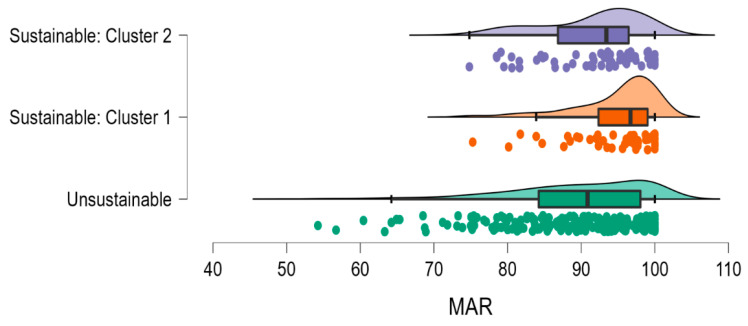
Raincloud plot showing raw data, density, boxplots, and means with 95% CI for the Mean Adequacy Ratio. *n* = 216, *n* = 54, and *n* = 57 for unsustainable, Cluster 1, and Cluster 2, respectively. MAR = Mean Adequacy Ratio. Those in Cluster 1 consumed a more nutrient-dense diet than those consuming an unsustainable diet (*p* < 0.001) or those in Cluster 2 (*p* < 0.01). MAR did not differ between those consuming an unsustainable diet and those in Cluster 2 (*p* = 0.851).

Compared to those in cluster 1, those in cluster 2 failed to meet the Reference Nutrient Intake (RNI) for folate (X^2^ (1, 111) = 4.198, *p* < 0.037), selenium (X^2^ (1, 111) = 33.466, *p* < 0.001), zinc (X^2^ (1, 111) = 20.229 < 0.001), and protein (X^2^ (1, 111) = 8.950, *p* < 0.002). Levels of iron were borderline (X^2^ (1, 111) =3.242, *p* = 0.072) (Table 2). There were no differences between clusters regarding calcium (X^2^ (1, 111) = 0.881, *p* = 0.238), copper (X^2^ (1, 111) = 0.003, *p* = 0.557), magnesium (X^2^ (1, 111) = 0.259, *p* = 0.382), iodine (X^2^ (1, 111) = 1.366, *p* = 0.165), or B12 (X^2^ (1, 111) = 1.788, *p* = 0.167). There was also a significant difference in the Mean Adequacy Ratio (MAR) between groups (*F* (1, 109) = 6.746, *p* < 0.010, η^2^ = 0.059; Figure 3).

### 3.5. Association between Healthy and Unhealthy Sustainable Dietary Patterns and Health Outcomes

Those in cluster 2 had a more depressed mood (*F* (1, 109) = 6.366, *p* < 0.013, η^2^ = 0.056). However, there was no association with HRV (*F* (1, 109) = 1.319, *p* = 0.253, η^2^ = 0.012).

## 4. Discussion

The present study aimed to determine the association between the EAT–Lancet diet and cognitive and affective health. Key findings were: (1) in this sample of young adults, adherence to the EAT–Lancet diet was low; (2) after controlling for various confounds, adherence to the EAT–Lancet diet was associated with a less depressed mood and more accurate focused attention, although the effect was less than with the AHEI, which, in addition, was also associated with better episodic memory and higher heart rate variability; (3) those who adhered strongly to the recommendation to limit red meat and poultry (*n* = 57) did not reach the RNI for essential nutrients that are important for the brain, including protein, selenium, zinc, iron, and folate. Finally, (4) this cluster of individuals with a limited intake of meat and poultry also reported a poorer mood. These data suggested that advocating a strict adoption of the EAT–Lancet diet might not be optimal for brain functioning.

Regarding adherence to the EAT–Lancet diet, the present data agree with previous findings [1,31]. Most of this sample of young adults living in the UK did not adhere to a sustainable diet as proposed by the EAT–Lancet Commission. As shown in Figure 1, this population tended to overconsume the high-environmental-impact foods red meat (71% non-adherence) and poultry (66% non-adherence). Although whole grains were considered a low environmental impact and a protective food [31], the present sample consumed on average 125 g/day more that the recommended intake. Under consumed foods included nuts, with 96% of the sample consuming < the minimum recommended intake, and dairy, with 100% of the sample consuming <50% of the maximum recommended intake. A total of 23% of the sample consumed less than the lower recommended intake for vegetables, and 31% consumed less than the recommended intake for fruit (Figure 1). Overall, these findings highlight a need to improve the sustainability of the UK young-adult diet with a particular focus on red meat and poultry. However, as detailed below, this may present major challenges in terms of ensuring adequate nutrient intake for the brain.

Across the entire sample, adherence to the EAT–Lancet diet was associated with a less-depressed mood and more-focused attention, although no effects were observed for heart rate variability or memory. This is consistent with existing evidence that plant-based diets are associated with a range of positive health outcomes [57,58]. Importantly, these effects were observed after controlling for gender, BMI, total calories, physical activity, and alcohol consumption. However, we also observed that these beneficial effects were often larger when the Alternative Healthy Eating Index (AHEI) was considered. Furthermore, whereas adherence to the AHEI was positively associated with episodic and working memory performance and HRV, adherence to the WISH was not. One explanation for a smaller benefit of the EAT–Lancet diet is the recommendation to limit animal products, with minimum intake values of 0 g/d. For example, previously this was associated with the decreased probability of micronutrient adequacy, including calcium, folate, iron, vitamin A, vitamin C, and zinc [18]. Many of these nutrients are essential for the brain [20,21,22,23,24,25,26,27].

Indeed, herein we identified a cluster of individuals, a sixth of the present sample, who were more at risk of being deficient in a range of nutrients, including protein, selenium, zinc, iron, and folate (Table 2), and had a lower Mean Adequacy Ratio (Figure 3). More specifically, two dietary patterns in the highest tertile of the planetary health diet (*n* = 111) were identified. Both clusters of individuals were characterised by high adherence to the EAT–Lancet recommendations for low-environmental-impact foods, i.e., vegetables, whole grains, and added sugar; moderate adherence to recommendations for fruit, legumes, dairy, eggs, and saturated fats; and low adherence to the recommendation for nuts. However, whereas those in cluster 1 (*n* = 54) adhered strongly to the recommendation to consume moderate amounts of fish and unsaturated fats, they did not adhere to the recommendation to *limit* poultry and red meat—in fact, consumption of these food items was moderate to high. Conversely, cluster 2 (*n* = 57) scored lower in their adherence to fish and unsaturated oils (72% consuming less than the minimum recommended intake); however, they adhered strongly to the recommendation to *limit* poultry and red meat (note that cluster 2 did not represent the vegan diet, as 98.7% of the group reported consuming at least some animal products, with 50% consuming >200 g/d) (Figure 2).

Notably, those in cluster 2 also reported higher levels of negative affect. Whilst we cannot determine causality, this finding raises the possibility that the multiple nutrient deficiencies associated with this style of eating may have consequences for brain functioning. Indeed, one of the important roles of dietary protein is to provide a supply of amino acids to produce neurotransmitters, such as catecholamines and serotonin. Tryptophan and phenylalanine are essential amino acids that are implicated in the development of mood disorders [59,60]. Importantly, the effect on mood observed herein is consistent with previous observations that those who abstain from meat consumption had higher rates of depression [20,61,62]. Data from the UK Biobank cohort indicated that vegetarians had low levels of B6 and B12 and a higher prevalence of depression [63]. Furthermore, vegan diets, in which all animal products are restricted, were associated with a low intake of vitamins B_2_, niacin (B_3_), B_12_, D, iodine, zinc, calcium, potassium, selenium, and protein [19]. Similarly, a Danish study observed that the EAT–Lancet diet was deficient in calcium, zinc, iodine, and selenium [64]. Taken together with the present findings, this literature suggests that strict adherence to the EAT–Lancet recommendation to limit red meat and poultry may reduce the nutrient density of some people’s diet, with consequences for affect. Unfortunately, due to sample-size constraints, it was not possible to consider effects on cognition, but this will be an important area for future research.

Although the dietary clusters identified herein require replication in a larger sample, they emphasised a need to design sustainable diets that are both healthy for the brain and acceptable to the relevant sub-population. Unfortunately, when designing sustainable diets that are also healthy, there has been a tendency to focus on evidence concerning physiological endpoints such as cancer, cardiovascular disease, diabetes, and/or overall mortality [1]. Cognitive and affective health has either been ignored or discounted as only being relevant to specific sub-populations. Nonetheless, the present findings suggest that, for some individuals, a shift towards sustainable eating may negatively influence brain functioning. Therefore, a broader consideration of health, including behavioural outcomes, is warranted.

In the present sample, the consumption of nuts was well below the amount recommended by the EAT–Lancet Commission. Nuts and seeds are a rich source of selenium (an antioxidant mineral utilized in selenoproteins important for brain functioning [65]) and an important source of folate, zinc, and protein. As those in cluster 2 were deficient in these nutrients, it is tempting to suggest that they would benefit from eating more nuts and seeds, which would bring them more in line with the EAT–Lancet reference diet and increase the Mean Adequacy Ratio in this group. Notably, in the Spanish PREDIMED trial, those who consumed 30 g of mixed nuts per day as part of a Mediterranean diet experienced a lower incidence of cardiovascular disease and cognitive decline [66,67]. However, it needs to be considered that dietary recommendations that deviate dramatically from what is normally consumed are less likely to be acceptable or achievable [68]. Given that nuts and seeds are readily available in the UK, one can assume that, in the present sample, these foods were not preferred.

The development of alternative proteins, for example, algae, insects, cultured meat, and plant-based alternatives might present one viable solution, although they are likely to encounter similar consumer resistance [69]. In addition, except for plant-based proteins such as pulses [70,71], there is currently little or no research assessing the cognitive or affective consequences of these ‘alternative’ foods. In the alternative protein market, there also tends to be a focus on crude protein content. However, as the present data illustrate, animal proteins provide more than just protein (i.e., micronutrients), and the bioavailability of these nutrients in alternative proteins needs to be established. There is also a need for more research to identify the nutritional equivalence of alternative protein sources compared to animal-derived proteins. For example, a recent metabolomics study compared the nutritional composition of a plant-based meat alternative with grass-fed animal meat. The products had almost identical macro- nutrients, and 22 metabolites were found exclusively in the meat, whilst 31 metabolites were found to be exclusive to the plant-based product [72]. Hence, products may differ in ways that are currently unknown, and consumers choosing to eliminate one or the other product could miss out on key nutrients [72].

Finally, the identification of a cluster of individuals whose diet was sustainable yet unhealthy for the brain emphasised a need for future research to identify individual differences in the propensity to adopt this dietary style. Herein, we found that being female and consuming fewer calories were two potential risk factors suggesting that restrained or restricted eating styles might be important. Other risk factors that could prove profitable include food fussiness/neophobia, education level/nutrition knowledge, ethnicity, food insecurity, ethical and health motivations, and compulsivity and/or orthorexic behaviours.

This study has both strengths and limitations. We had an ample sample size to detect meaningful associations between dietary style and mood and HRV. However, the sample was reduced for cognition; therefore, replication in larger cohorts will be necessary. Our sample consisted of young healthy adults, and whilst data indicate that this population is the most likely to adopt meat-free diets [73], other populations may be more at risk of deficiency. For example, older individuals may require more protein, as low protein intake was associated with an increase in frailty [74]. Therefore, determining associations between the EAT–Lancet diet and cognitive decline in older individuals will be important. Whilst the weaknesses of food frequency questionnaires were recognised [75], the EPIC-Norfolk FFQ has been validated against a 16-day weighed food record [48] and nutrient biomarkers [49]. Although we controlled for a range of potential confounds, the cross-sectional design means that causality cannot be determined. There is an urgent need for large randomised controlled trials assessing the psychological effects of novel sustainable foods and dietary patterns.

Our use of the UK RNIs to determine nutrient adequacy should not be interpreted as suggesting that those adhering to the EAT–Lancet Commission’s recommendation to limit meat intake *are* deficient in these nutrients. Rather the present data suggested that these individuals are at a greater *risk* of nutrient deficiency, as their intake of specific nutrients were below that considered adequate for 95% of the UK population. Studies that incorporate nutrient biomarkers and clinical signs of deficiency will be needed to determine the accurate prevalence of nutrient deficiency.

## 5. Conclusions

In conclusion, this study is the first to determine the effects of the EAT–Lancet reference diet on cognition, mood, and HRV. The results indicated that, compared to the AHEI, adherence to a sustainable dietary pattern was less likely to be beneficial. That is, adherence to the AHEI was associated with having a better mood, focused attention, memory, and higher heart rate variability. However, when the EAT–Lancet diet was considered, the effects were either smaller or absent. Cluster analysis identified a group of individuals who adhered strongly to the recommendation to limit meat intake; these individuals reported a poorer mood and were at risk of being deficient in a range of nutrients, including protein, selenium, zinc, iron, and folate, contributing to a lower Mean Adequacy Ratio (Figure 3). These data highlighted the potential unintended consequences of the EAT–Lancet diet for affective health in some individuals. Randomised controlled trials and computational studies are urgently required to determine whether it is possible to better optimise sustainable diets to support brain health.

## Figures and Tables

**Table 1:** WISH dietary components contributing to each cluster.

Food Group	Cluster 1: Balanced (N = 54)	Cluster 2: Restricted (N = 57)	*F*	*p*
Vegetables	9.38 (0.35)	5.52 (0.34)	4.477	0.037
Fruit	7.35 (0.53)	7.76 (0.51)	0.243	0.623
Whole grains	9.54 (0.29)	9.92 (0.28)	0.322	0.572
Legumes	5.74 (0.45)	6.63 (0.43)	0.069	0.793
Unsaturated fats	6.45 (0.52)	2.25 (0.50)	30.487	<0.001
Nuts	1.33 (0.18)	1.17 (0.17)	0.035	0.853
Fish	8.72 (0.50)	4.04 (0.48)	51.476	<0.001
Dairy	5.61 (0.35)	5.58 (0.33)	0.795	0.375
Saturated fats	6.41 (0.62)	7.75 (0.59)	10.448	0.002
Sugar	9.81 (0.18)	9.82 (0.17)	0.001	0.970
Red meat	2.26 (0.55)	8.27 (0.38)	155.136	<0.001
Eggs	8.79 (0.38)	9.18 (0.36)	2.056	0.155
Poultry	1.73 (0.33)	9.82 (0.32)	82.225	<0.001
BMI (kg/m^2^)	25.83 (0.76)	25.54 (0.72)	0.074	0.786
Alcohol (g)	6.91 (1.04)	4.33 (1.00)	3.187	0.077
Exercise (min/week)	130.90 (5.80)	118.69 (5.54)	2.316	0.131
Kcal	1850.57 (69.57)	1671.54 (66.5)	3.460	0.066
Mood	2.05 (0.18)	2.63 (0.17)	5.594	0.020
HF HRV	55.41 (3.44)	50.24 (3.25)	1.126	0.291

Unless otherwise stated, data are means (SEM) of the WISH score for each dietary component. BMI—Body mass index. HF HRV—High-frequency heart rate variability.

**Table 2:** Percentage of those in the two dietary clusters in the top tertial of the EAT–Lancet diet who consumed nutrient intakes below the RNI.

Nutrient	Unsustainable (n = 215)	Cluster 1: Balanced (n = 54)	Cluster 2: Restricted (n = 57)	*X*^2^ Cluster 1 vs. Cluster 2	*p* Cluster 1 vs. Cluster 2
Calcium	35.5%	26.4%	19.0%	0.881	0.238
Copper	44.2%	30.0%	34.5%	0.003	0.557
Iron	77.0%	69.8%	84.2%	3.242	0.072
Folate	91.2%	77.4%	91.4%	4.198	0.037
Iodine	61.1%	34.0%	44.8%	1.366	0.165
Magnesium	60.8%	28.3%	32.8%	0.259	0.382
Selenium	50.7%	22.6%	77.6%	33.466	0.001
Zinc	65.8%	41.5%	82.8%	20.229	0.001
B12	8.3%	3.8%	10.3%	1.788	0.167
Protein	7.2%	0.0%	15.5%	8.950	0.002
MAR	89.43(0.58)	95.01(1.19)	91.66(1.12)	9.254	0.001

Nutrient adequacy was based on the UK government’s dietary recommendations for energy and nutrients for males and females aged 19+ years [56]. MAR—Mean Adequacy Ratio.

## Data Availability

Upon publication, data will be made available at https://doi.org/10.5061/dryad.1jwst, accessed on 17 September 2022.

## Supplementary Materials

The following supporting information can be downloaded at: https://www.mdpi.com/article/10.3390/nu14204254/s1, Table S1. Construction of the AHEI-2010 scoring method. Table S2. Construction of the WISH diet score [1,31,76,77,78]. Figure S1. Illustration of the procedure.

## References

[1] Willett W.J., Rockström B., Loken M., Springmann T., Lang S., Vermeulen T., Garnett D., Tilman F., DeClerck A., Wood M. (2019). Food in the Anthropocene: The EAT–Lancet Commission on healthy diets from sustainable food systems. Lancet.

[2] World Health Organization (2019). Sustainable Healthy Diets: Guiding Principles.

[3] Zagmutt F.J., Pouzou J.G., Costard S. (2020). The EAT-Lancet Commission’s dietary composition may not prevent noncommunicable disease mortality. J. Nutr..

[4] Zagmutt F.J., Pouzou J.G., Costard S. (2019). The EAT–Lancet Commission: A flawed approach?. Lancet.

[5] Harcombe Z. (2020). This is not the EAT–Lancet Diet. Lancet.

[6] Kaiser M. (2021). What is wrong with the EAT Lancet report?. Justice and Food Security in a Changing Climate.

[7] Young H.A., Benton D. (2018). Heart-rate variability: A biomarker to study the influence of nutrition on physiological and psychological health?. Behav. Pharmacol..

[8] Katz D., Meller S. (2014). Can We Say What Diet Is Best for Health?. Annu. Rev. Public Health.

[9] Qian F., Liu G., Hu F.B., Bhupathiraju S.N., Sun Q. (2019). Association Between Plant-Based Dietary Patterns and Risk of Type 2 Diabetes: A Systematic Review and Meta-analysis. JAMA Intern. Med..

[10] Patel H., Chandra S., Alexander S., Soble J., Williams K.A. (2017). Plant-Based Nutrition: An Essential Component of Cardiovascular Disease Prevention and Management. Curr. Cardiol. Rep..

[11] Kahleova H., Levin S., Barnard N. (2017). Cardio-Metabolic Benefits of Plant-Based Diets. Nutrients.

[12] Walker M.E., O’Donnell A.A., Himali J.J., Rajendran I., van Lent D.M., Ataklte F., Jacques P.F., Beiser A.S., Seshadri S., Vasan R.S. (2021). Associations of the Mediterranean-Dietary Approaches to Stop Hypertension Intervention for Neurodegenerative Delay diet with cardiac remodelling in the community: The Framingham Heart Study. Br. J. Nutr..

[13] Knuppel A., Papier K., Key T.J., Travis R.C. (2019). EAT-Lancet score and major health outcomes: The EPIC-Oxford study. Lancet.

[14] Ibsen D.B., Christiansen A.H., Olsen A., Tjønneland A., Overvad K., Wolk A., Mortensen J.K., Dahm C.C. (2022). Adherence to the EAT-Lancet Diet and Risk of Stroke and Stroke Subtypes: A Cohort Study. Stroke.

[15] Stubbendorff A., Sonestedt E., Ramne S., Drake I., Hallström E., Ericson U. (2021). Development of an EAT-Lancet index and its relation to mortality in a Swedish population. Am. J. Clin. Nutr..

[16] Gijsbers L., Ding E.L., Malik V.S., de Goede J., Geleijnse J.M., Soedamah-Muthu S.S. (2016). Consumption of dairy foods and diabetes incidence: A dose-response meta-analysis of observational studies. Am. J. Clin. Nutr..

[17] Petti A., Palmieri B., Vadalà M., Laurino C. (2017). Vegetarianism and veganism: Not only benefits but also gaps. A review. Prog. Nutr..

[18] Hanley-Cook G.T., Argaw A.A., de Kok B.P., Vanslambrouck K.W., Toe L.C., Kolsteren P.W., Jones A.D., Lachat C.K. (2021). EAT–Lancet diet score requires minimum intake values to predict higher micronutrient adequacy of diets in rural women of reproductive age from five low- and middle-income countries. Br. J. Nutr..

[19] Bakaloudi D.R., Halloran A., Rippin H.L., Oikonomidou A.C., Dardavesis T.I., Williams J., Wickramasinghe K., Breda J., Chourdakis M. (2021). Intake and adequacy of the vegan diet. A systematic review of the evidence. Clin. Nutr..

[20] Iguacel I., Huybrechts I., A Moreno L., Michels N. (2020). Vegetarianism and veganism compared with mental health and cognitive outcomes: A systematic review and meta-analysis. Nutr. Rev..

[21] Tardy A.-L., Pouteau E., Marquez D., Yilmaz C., Scholey A. (2020). Vitamins and Minerals for Energy, Fatigue and Cognition: A Narrative Review of the Biochemical and Clinical Evidence. Nutrients.

[22] Grosso G., Pajak A., Marventano S., Castellano S., Galvano F., Bucolo C., Drago F., Caraci F. (2014). Role of Omega-3 Fatty Acids in the Treatment of Depressive Disorders: A Comprehensive Meta-Analysis of Randomized Clinical Trials. PLoS ONE.

[23] Sandstead H.H. (2000). Causes of Iron and Zinc Deficiencies and Their Effects on Brain. J. Nutr..

[24] Yurko-Mauro K., Alexander D.D., Van Elswyk M.E. (2015). Docosahexaenoic Acid and Adult Memory: A Systematic Review and Meta-Analysis. PLoS ONE.

[25] Marchetti M.F., da Silva G.M., Freiria C.N., Borim F.S.A., de Brito T.R.P., Milanski M., Corona L.P. (2022). Association between zinc deficiency and cognitive decline in community-dwelling older adults. Ciência Saúde Coletiva.

[26] Vogel T., Dali-Youcef N., Kaltenbach G., Andrès E. (2009). Homocysteine, vitamin B_12_, folate and cognitive functions: A systematic and critical review of the literature. Int. J. Clin. Pract..

[27] Fairbairn P., Dyall S., Tsofliou F. (2022). The Effects of Multi-Nutrient Formulas containing a Combination of Omega-3 Polyunsaturated Fatty Acids and B vitamins on Cognition in the older adult: A Systematic Review and Meta-analysis. Br. J. Nutr. Int. J. Nutr. Sci..

[28] Georgieff M.K. (2007). Nutrition and the developing brain: Nutrient priorities and measurement. Am. J. Clin. Nutr..

[29] Black M.M. (2008). Effects of Vitamin B_12_ and Folate Deficiency on Brain Development in Children. Food Nutr. Bull..

[30] Dalile B., Kim C., Challinor A., Geurts L., Gibney E.R., Galdos M.V., La Fata G., Layé S., Mathers J.C., Vauzour D. (2022). The EAT–Lancet reference diet and cognitive function across the life course. Lancet Planet. Health.

[31] Trijsburg L., Talsma E., Crispim S., Garrett J., Kennedy G., de Vries J., Brouwer I. (2020). Method for the Development of WISH, a Globally Applicable Index for Healthy Diets from Sustainable Food Systems. Nutrients.

[32] Cacau L.T., De Carli E., de Carvalho A.M., Lotufo P.A., Moreno L.A., Bensenor I.M., Marchioni D.M. (2021). Development and Validation of an Index Based on EAT-Lancet Recommendations: The Planetary Health Diet Index. Nutrients.

[33] Gallagher C.T., Hanley P., Lane K.E. (2021). Pattern analysis of vegan eating reveals healthy and unhealthy patterns within the vegan diet. Public Health Nutr..

[34] Rajaram S., Jones J., Lee G.J. (2019). Plant-based dietary patterns, plant foods, and age-related cognitive decline. Adv. Nutr..

[35] Young H., Benton D. (2015). We should be using nonlinear indices when relating heart-rate dynamics to cognition and mood. Sci. Rep..

[36] Young H., Benton D., Carter N. (2015). The Effect of Chicken Extract on Mood, Cognition and Heart Rate Variability. Nutrients.

[37] Young H.A., Cousins A., Johnston S., Fletcher J.M., Benton D. (2019). Autonomic adaptations mediate the effect of hydration on brain functioning and mood: Evidence from two randomized controlled trials. Sci. Rep..

[38] Young H.A., Cousins A.L., Watkins H.T., Benton D. (2017). Is the link between depressed mood and heart rate variability explained by disinhibited eating and diet?. Biol. Psychol..

[39] Young H.A., Davies J., Freegard G., Benton D. (2021). Nonsuicidal Self-Injury Is Associated with Attenuated Interoceptive Responses to Self-Critical Rumination. Behav. Ther..

[40] Young H.A., Gaylor C.M., De Kerckhove D., Watkins H., Benton D. (2019). Interoceptive accuracy moderates the response to a glucose load: A test of the predictive coding framework. Proc. R. Soc. B Boil. Sci..

[41] Young H.A., Watkins H. (2016). Eating disinhibition and vagal tone moderate the postprandial response to glycemic load: A randomized controlled trial. Sci. Rep..

[42] Young H.A., Williams C., Pink A.E., Freegard G., Owens A., Benton D. (2017). Getting to the heart of the matter: Does aberrant interoceptive processing contribute towards emotional eating?. PLoS ONE.

[43] Mulligan A.A., Luben R.N., Bhaniani A., Parry-Smith D.J., O’Connor L., Khawaja A.P., Forouhi N.G., Khaw K.-T. (2014). A new tool for converting food frequency questionnaire data into nutrient and food group values: FETA research methods and availability. BMJ Open.

[44] Lorr M., McNair D.M., Fisher S. (1982). Evidence for bipolar mood states. J. Personal. Assess..

[45] Watson D., Clark L.A., Tellegen A. (1988). Development and validation of brief measures of positive and negative affect: The PANAS scales. J. Personal. Soc. Psychol..

[46] Fortier I., Raina P., Heuvel E.R.V.D., Griffith L.E., Craig C., Saliba M., Doiron D., Stolk R.P., Knoppers B.M., Ferretti V. (2017). Maelstrom Research guidelines for rigorous retrospective data harmonization. Int. J. Epidemiol..

[47] Wey T.W., Doiron D., Wissa R., Fabre G., Motoc I., Noordzij J.M., Ruiz M., Timmermans E., van Lenthe F.J., Bobak M. (2021). Overview of retrospective data harmonisation in the MINDMAP project: Process and results. J. Epidemiol. Community Health.

[48] Bingham S.A., Gill C., Welch A., Day K., Cassidy A., Khaw K., Sneyd M., Key T., Roe L., Day N. (1994). Comparison of dietary assessment methods in nutritional epidemiology: Weighed records v. 24 h recalls, food-frequency questionnaires and estimated-diet records. Br. J. Nutr..

[49] Bingham S.A., Welch A.A., McTaggart A., Mulligan A.A., Runswick S.A., Luben R., Oakes S., Khaw K.T., Wareham N., Day N.E. (2001). Nutritional methods in the European Prospective Investigation of Cancer in Norfolk. Public Health Nutr..

[50] Paul A., Southgate D. (1987). McCance and Widdowson’s the Composition of Foods. European Food Composition Tables in Translation.

[51] McCullough M.L., Feskanich D., Stampfer M.J., Giovannucci E.L., Rimm E.B., Hu F.B., Spiegelman D., Hunter D.J., A Colditz G., Willett W.C. (2002). Diet quality and major chronic disease risk in men and women: Moving toward improved dietary guidance. Am. J. Clin. Nutr..

[52] Wang D.D., Leung C.W., Li Y., Ding E., Chiuve S., Hu F.B., Willett W.C. (2014). Trends in Dietary Quality Among Adults in the United States, 1999 Through 2010. JAMA Intern. Med..

[53] Estrella M.L., A Durazo-Arvizu R., Mattei J., Mossavar-Rahmani Y., Perreira K.M., Siega-Riz A.M., Sotres-Alvarez D., González H.M., Gallo L.C., Daviglus M.L. (2020). Alternate Healthy Eating Index is Positively Associated with Cognitive Function Among Middle-Aged and Older Hispanics/Latinos in the HCHS/SOL. J. Nutr..

[54] Tomova G.D., Arnold K.F., Gilthorpe M.S., Tennant P.W.G. (2021). Adjustment for energy intake in nutritional research: A causal inference perspective. Am. J. Clin. Nutr..

[55] Tarvainen M.P., Niskanen J.-P., Lipponen J.A., Ranta-Aho P.O., Karjalainen P.A. (2014). Kubios HRV–heart rate variability analysis software. Comput. Methods Programs Biomed..

[56] Recommendations G.D. (2016). Government Recommendations for Energy and Nutrients for Males and Females Aged 1–18 Years and 19+ Years. Public Health Engl..

[57] Angelino D., Godos J., Ghelfi F., Tieri M., Titta L., Lafranconi A., Marventano S., Alonzo E., Gambera A., Sciacca S. (2019). Fruit and vegetable consumption and health outcomes: An umbrella review of observational studies. Int. J. Food Sci. Nutr..

[58] Godos J., Currenti W., Angelino D., Mena P., Castellano S., Caraci F., Galvano F., Del Rio D., Ferri R., Grosso G. (2020). Diet and Mental Health: Review of the Recent Updates on Molecular Mechanisms. Antioxidants.

[59] Lieberman H.R., Committee of Military Nutrition Research and Institute of Medicine (1999). Amino acid and protein requirements: Cognitive performance, stress and brain function. The Role of Protein and Amino Acids in Sustaining and Enhancing Performance.

[60] Strasser B., Gostner J.M., Fuchs D. (2016). Mood, food, and cognition: Role of tryptophan and serotonin. Curr. Opin. Clin. Nutr. Metab. Care.

[61] Dobersek U., Wy G., Adkins J., Altmeyer S., Krout K., Lavie C.J., Archer E. (2021). Meat and mental health: A systematic review of meat abstention and depression, anxiety, and related phenomena. Crit. Rev. Food Sci. Nutr..

[62] Ocklenburg S., Borawski J. (2021). Vegetarian diet and depression scores: A meta-analysis. J. Affect. Disord..

[63] Berkins S., Schiöth H., Rukh G. (2021). Depression and Vegetarians: Association between Dietary Vitamin B6, B12 and Folate Intake and Global and Subcortical Brain Volumes. Nutrients.

[64] Lassen A.D., Christensen L.M., Trolle E. (2020). Development of a Danish Adapted Healthy Plant-Based Diet Based on the EAT-Lancet Reference Diet. Nutrients.

[65] Schweizer U., Bohleber S., Zhao W., Fradejas-Villar N. (2021). The Neurobiology of Selenium: Looking Back and to the Future. Front. Neurosci..

[66] Martínez-Lapiscina E.H., Clavero P., Toledo E., Estruch R., Salas-Salvadó J., San Julián B., Sanchez-Tainta A., Ros E., Valls-Pedret C., Martinez-Gonzalez M.Á. (2013). Mediterranean diet improves cognition: The PREDIMED-NAVARRA randomised trial. J. Neurol. Neurosurg. Psychiatry.

[67] Estruch R., Ros E., Salas-Salvadó J., Covas M.-I., Corella D., Arós F., Gómez-Gracia E., Ruiz-Gutiérrez V., Fiol M., Lapetra J. (2018). Primary Prevention of Cardiovascular Disease with a Mediterranean Diet Supplemented with Extra-Virgin Olive Oil or Nuts. N. Engl. J. Med..

[68] Onwezen M.C., Bouwman E.P., Reinders M.J., Dagevos H. (2021). A systematic review on consumer acceptance of alternative proteins: Pulses, algae, insects, plant-based meat alternatives, and cultured meat. Appetite.

[69] Gazan R., Brouzes C.M.C., Vieux F., Maillot M., Lluch A., Darmon N. (2018). Mathematical Optimization to Explore Tomorrow’s Sustainable Diets: A Narrative Review. Adv. Nutr..

[70] Yeh T.-S., Yuan C., Ascherio A., A Rosner B., Blacker D., Willett W.C. (2021). Long-term dietary protein intake and subjective cognitive decline in US men and women. Am. J. Clin. Nutr..

[71] Mazza E., Fava A., Ferro Y., Moraca M., Rotundo S., Colica C., Provenzano F., Terracciano R., Greco M., Foti D. (2017). Impact of legumes and plant proteins consumption on cognitive performances in the elderly. J. Transl. Med..

[72] Van Vliet S., Bain J.R., Muehlbauer M.J., Provenza F.D., Kronberg S.L., Pieper C.F., Huffman K.M. (2021). A metabolomics comparison of plant-based meat and grass-fed meat indicates large nutritional differences despite comparable Nutrition Facts panels. Sci. Rep..

[73] Kamiński M., Skonieczna-Żydecka K., Nowak J.K., Stachowska E. (2020). Global and local diet popularity rankings, their secular trends, and seasonal variation in Google Trends data. Nutrition.

[74] Shekhar M., Rahnev D. (2021). Sources of Metacognitive Inefficiency. Trends Cogn. Sci..

[75] Briefel R.R., Flegal K.M., Winn D.M., Loria C.M., Johnson C.L., Sempos C.T. (1992). Assessing the nation’s diet: Limitations of the food frequency questionnaire. J. Am. Diet. Assoc..

[76] Chiuve S.E., Fung T.T., Rimm E.B., Hu F.B., McCullough M.L., Wang M., Stampfer M.J., Willett W.C. (2012). Alternative Dietary Indices Both Strongly Predict Risk of Chronic Disease. J. Nutr..

[77] Clark M.A., Springmann M., Hill J., Tilman D. (2019). Multiple health and environmental impacts of foods. Proc. Natl. Acad. Sci. USA.

[78] Afshin A., Sur P.J., Fay K.A., Cornaby L., Ferrara G., Salama J.S., Mullany E.C., Abate K.H., Cristiana A., Abebe Z. (2019). Health effects of dietary risks in 195 countries, 1990–2017: A systematic analysis for the Global Burden of Disease Study 2017. Lancet.

