# Supplementary material for: Adherence to the EAT–Lancet Diet: Unintended Consequences for the Brain?
Source: Nutrients. 2022 Oct 12;14(20):4254. doi: 10.3390/nu14204254 (PMC9607064; doi:10.3390/nu14204254)
Supplement: Supplementary file 1 [file nutrients-14-04254-s001.zip › Supplementary Figure S1 Procedure.pdf]

Figure S1. Schematic illustration of the experimental procedure.

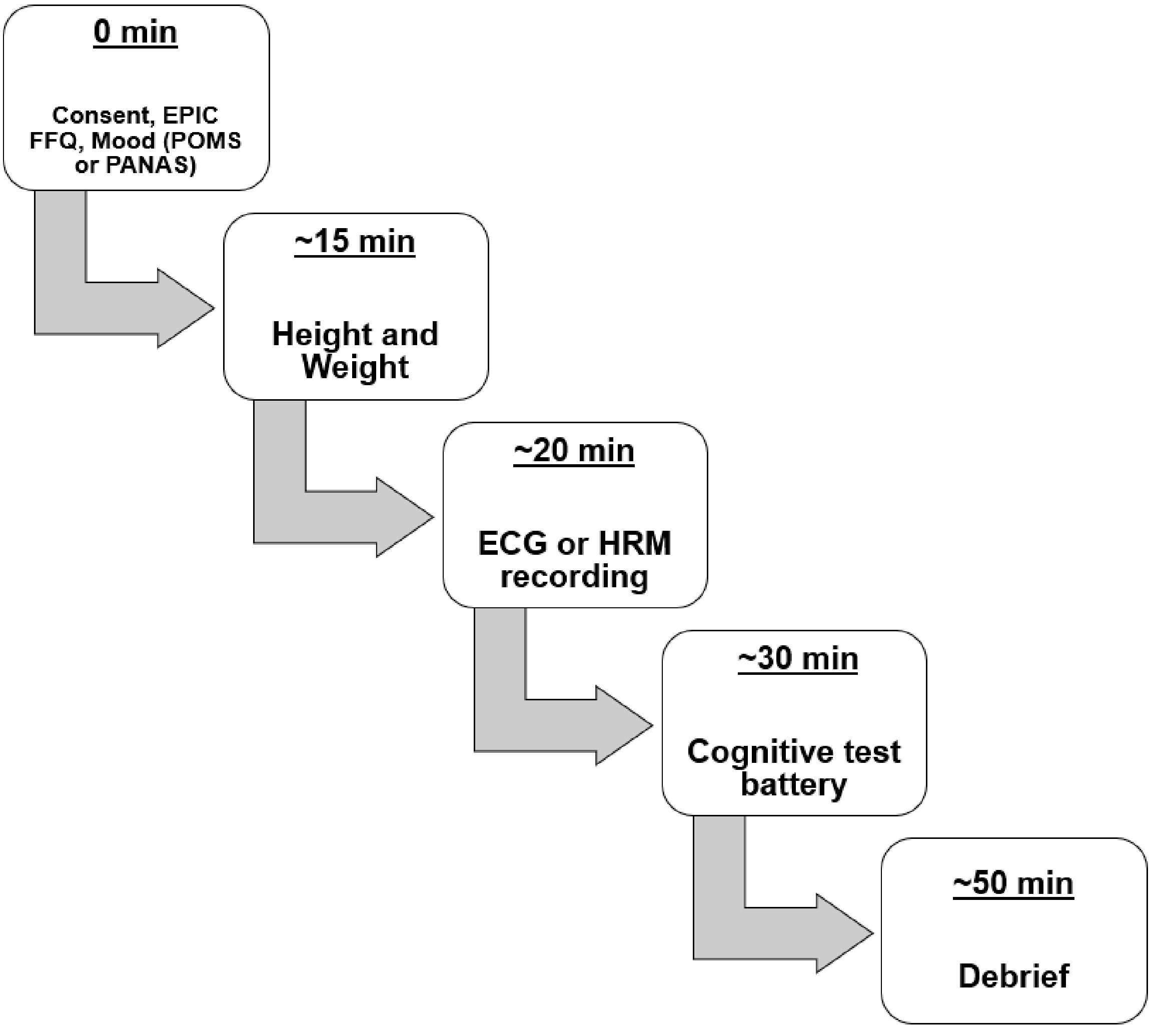

Note. EPIC FFQ - European Prospective Investigation into Cancer and Nutrition Norfolk Food Frequency Questionnaire, POMS – Profile of Mood States, PANAS – Positive and Negative Affect Scale, ECG – Electrocardiogram, HRM – Heart Rate Monitor.
