# Supplementary material for: Adherence to the EAT–Lancet Diet: Unintended Consequences for the Brain?
Source: Nutrients. 2022 Oct 12;14(20):4254. doi: 10.3390/nu14204254 (PMC9607064; doi:10.3390/nu14204254)
Supplement: Supplementary file 1 [file nutrients-14-04254-s001.zip › Supplementary Materials_dietary pattern construction.pdf]

Supplementary Table S1. Construction of the AHEI-2010 scoring method

Component	Criteria for minimum score (0)	Criteria for maximum score (10)
Vegetables, servings/d	0	≥5 (400g)
Fruit, servings/d	0	≥4 (320g)
Whole grains, g/d	0	
Women		75
Men		90
Sugar-sweetened beverages and fruit juice, servings/d	≥1 (230g)	0
Nuts and legumes, servings/d	0	≥1 (30g)
Red/processed meat, servings/d	≥1.5 (150g)	0
trans Fat, % of energy	≥4	≤0.5
Long-chain (n-3) fats (EPA + DHA), mg/d	0	250
PUFA, % of energy	≤2	≥10
Sodium, mg/d	Highest decile	Lowest decile
Alcohol, drinks/d		
Women	≥2.5	0.5–1.5
Men	≥3.5	0.5–2.0
Total	0	110

Note: for the purposes of the present study alcohol was not included in the calculation of the AHEI. Modified from Chiuve et al. (2012).

Supplementary Table S2. Construction of the WISH diet score

Component	Food items based on the food frequency questionnaire (FFQ)**	Healthiness[1]	Impact on Environment[2]	Recommended Intake in g/day (Lower and Upper Range of Intake)[3]
**Whole grains**	Brown bread, wholemeal bread, cream crackers, white bread, crispbread, porridge, breakfast cereal, white rice, brown rice, white pasta, wholemeal pasta, lasagne, pizza	Protective	Low	≥125 (100–150) [3]
**Vegetables**	Carrots, spinach, broccoli, leafy greens, brussels sprouts, cabbage, peas, green beans, courgettes, cauliflower, parsnips, leeks, onion, garlic, mushrooms, sweet peppers, beansprouts, salad vegetables, watercress, tomatoes, sweetcorn, beetroot, coleslaw, avocado	Protective	Low	300 (200–600)

**Fruits**	Apples, pears, oranges, grapefruit, bananas, grapes, melon, stone fruit, berries', kiwi fruit	Protective	Low	200 (100–300)
**Dairy foods**	Low fat yogurt, full fat yogurt, soft cheese, hard cheese, dairy milk	Protective	Medium	250 (0–500)
**Red meat**	Beef, beef burgers, pork, lamb, bacon, ham, luncheon meats, sausages, savoury pies, liver	Limit	High	14 (0–28)
**Fish**	Fried fish in batter, fish cakes, other white fish, oily fish, shellfish, fish roe	Protective	High	28 (0–100)
**Eggs**	Eggs (boiled, fried or scrambled, etc.)	Neutral	Medium	13 (0–25)
**Chicken and other poultry**	Chicken or other poultry	Neutral	Medium	29 (0–58)
**Legumes**	Dried lentils, beans, peas and baked beans, tofu, soya meat, TVP, vegeburger	Protective	Low	75 (0–100)
**Nuts**	Peanuts or other nuts, peanut butter	Protective	Medium	50 (0–75)
**Unsaturated oils**	Derived from the entire FFQ	Protective	Low	40 (20–80)

**Saturated oils**	Derived from the entire FFQ	Limit	High	11.8 (0–11.8)
**Added sugars**	Derived from the entire FFQ	Limit	Low	31 (0–31)

Note. The components included in the World Index for Sustainability and Health (WISH). [1] Based on the supplementary material of the EAT Lancet recommendations (Willett et al., 2019). [2] Based on the assessment of Clark et al. (2019) with sustainability indicators: greenhouse gas, land use, eutrophication, acidification, and scarcity weighted water. [3] Recommended amounts of intake obtained from Global Burden of Disease study ("Health effects of dietary risks in 195 countries, 1990-2017: a systematic analysis for the Global Burden of Disease Study 2017," 2019). **EPIC-Oxford FFQ can be found here http://www.epic-oxford.org/files/epic-baseline-PQ.pdf. Modified from Trijsburg et al. (2020).

References

Chiuve, S.E.; Fung, T.T.; Rimm, E.B.; Hu, F. B.; McCullough, M.L.; Wang, M.; Stampfer, M.J.; Willett, W.C. Alternative Dietary Indices Both Strongly Predict Risk of Chronic Disease. *J. Nutr.* **2012**, *142*, 1009–1018. https://doi.org/10.3945/jn.111.157222.

Clark, M.A.; Springmann, M.; Hill, J.; Tilman, D. Multiple health and environmental impacts of foods. *Proc. Natl. Acad. Sci. USA* **2019**, *116*, 23357-23362. https://doi.org/10.1073/pnas.1906908116.

Afshin, A.; Sur, P.J.; Fay, K.A.; Cornaby, L.; Ferrara, G.; Salama, J.S.; Mullany, E.C.; Abate, K.H.; Cristiana, A.; Abebe, Z.; et al. Health effects of dietary risks in 195 countries, 1990–2017: A systematic analysis for the Global Burden of Disease Study 2017. *Lancet* **2019**, *393*, 1958–1972. https://doi.org/10.1016/s0140-6736(19)30041-8.

Trijsburg, L.; Talsma, E.F.; Crispim, S.P.; Garrett, J.; Kennedy, G.; de Vries, J.H.M.; Brouwer, I.D. Method for the Development of WISH, a Globally Applicable Index for Healthy Diets from Sustainable Food Systems. *Nutrients* **2020**, *13*, 93. https://doi.org/10.3390/nu13010093

Willett, W.; Rockström, J.; Loken, B.; Springmann, M.; Lang, T.; Vermeulen, S.; Garnett, T.; Tilman, D.; DeClerck, F.; Wood, A. (). Food in the Anthropocene: the EAT–Lancet Commission on healthy diets from sustainable food systems. *Lancet*, **2019**, *393*, 447–492.
